# Corrosion Behavior in Magnesium-Based Alloys for Biomedical Applications

**DOI:** 10.3390/ma15072613

**Published:** 2022-04-01

**Authors:** Liming Xu, Xingwang Liu, Kang Sun, Rao Fu, Gang Wang

**Affiliations:** 1 Institute of Materials, Shanghai University, Shanghai 200444, China; xlm123@shu.edu.cn (L.X.); g.wang@shu.edu.cn (G.W.); 2 Sports Medicine Department of Huashan Hospital, Fudan University, Shanghai 200040, China; 3 Department of Neurology, Tongren Hospital, School of Medicine, Shanghai Jiao Tong University, Shanghai 200336, China; fr4233@shtrhospital.com

**Keywords:** magnesium-based alloys, biodegradability, corrosion behavior, corrosive mechanism, corrosion resistance

## Abstract

Magnesium alloys exhibit superior biocompatibility and biodegradability, which makes them an excellent candidate for artificial implants. However, these materials also suffer from lower corrosion resistance, which limits their clinical applicability. The corrosion mechanism of Mg alloys is complicated since the spontaneous occurrence is determined by means of loss of aspects, e.g., the basic feature of materials and various corrosive environments. As such, this study provides a review of the general degradation/precipitation process multifactorial corrosion behavior and proposes a reasonable method for modeling and preventing corrosion in metals. In addition, the composition design, the structural treatment, and the surface processing technique are involved as potential methods to control the degradation rate and improve the biological properties of Mg alloys. This systematic representation of corrosive mechanisms and the comprehensive discussion of various technologies for applications could lead to improved designs for Mg-based biomedical devices in the future.

## 1. Introduction

Biomedical implants are typically composed of non-degradable metals, including stainless steel, titanium, and cobalt–chromium alloys. Degradable metals, such as magnesium, calcium, and zinc-based alloys, are also used. Non-degradable (permanent) materials have been widely used in biomedical applications for decades but exhibit certain limitations and challenges. For example, in orthopedics, the mismatched Young’s modulus between implants and surrounding tissue can produce serious stress-shielding, potentially causing unexpected bone atrophy [1]. The retained bulky implants can also irritate surrounding tissue and alter the forces and pressure on a joint complex [2]. The possibility of toxic ion release is another drawback of using permanent metals [3,4,5,6,7,8], which should be prevented to avoid unnecessary inflammation and infection.

In addition, traditional implants generate significantly more artifacts in common imaging modalities (e.g., digital radiography, magnetic resonance imaging, and computed tomography) and can create more artifact-induced noise and signal distortion in postoperative imaging [9,10]. Prior research has attempted to overcome these limitations, the most effective of which is the utilization of biodegradable metallic devices. Due to their inherent bioactive properties, the corresponding degradation products can actually improve the healing process in local tissue. As such, it is vitally important to develop practical degradable metals to meet clinical requirements.

Magnesium (Mg), the fourth most abundant cation in the human body, is mostly stored in the bone (60~65%) and soft tissues (35~40%, and mainly ~27% in the muscle) [11,12]. The U.S. Food and Nutrition Board recently established a recommended dietary allowance for magnesium of 420 mg/day for males and 320 mg/day for females [13]. Excess Mg ions can be stored in intracellular matrices, new bone formations, or dispersed into the circulatory system. This is often followed by excretion in urine to prevent hyper-magnesium [14,15]. When the degradation rate of an implanted device matches human metabolic capacity, a second surgical process (to remove the device) can typically be avoided. This can substantially reduce costs to healthcare providers and trauma to patients [13,16,17].

Mg also plays an important role in the synthesis of proteins and nucleic acid, the integrity of mitochondria and enzymes, the stabilization of biological membranes, and the modulation of transport functions [12,18,19]. The elastic modulus of Mg is ~45 GPa [20,21,22,23], which is close to the value of cancellous bone (10–30 GPa). Mg is also lightweight and exhibits a high strength-to-weight ratio. These suitable mechanical properties make Mg-based alloys a popular choice for medical devices requiring high biocompatibility, such as prosthetics and artificial implants [24,25]. However, these materials also exhibit a lower corrosion resistance, which leads to increased degradation rates. This can result in a premature loss of mechanical strength that limits the clinical applicability of these devices.

The investigation of medical Mg-based alloys began in 1878. However, the fundamental issues hindering its extensive application still remain. Specifically, higher chemical reactivity in physiological (pH = 7.4–7.6) environments leads to rapid degradation and subsequent implant failure [26,27,28]. This process is likely to induce a local accumulation of alkaline ions, causing cytotoxic effects and inflammation [29,30,31]. The production of hydrogen (H_2_) gas during degradation is another critical issue [32,33], which can result in a ballooning effect [30,34]. These accumulated gas bubbles can block normal blood flow, disrupt the balance of blood cell parameters, damage adjacent tissue, and cause infection [35,36,37,38]. A continued excess of Mg^2+^ may also decrease the crystallinity of hydroxyapatite (HA) and inhibit mineralization during the early phases of embryonic development [18]. Localized corrosion is another concern for the clinical application of Mg alloys. It can result from the formation of galvanic cells [39], high-strain regions [22], or corrosive conditions [31] that contribute to a rapid reduction in mechanical integrity [40,41]. These issues are the primary factors limiting the use of Mg-based devices in clinical settings.

**Figure 1:**
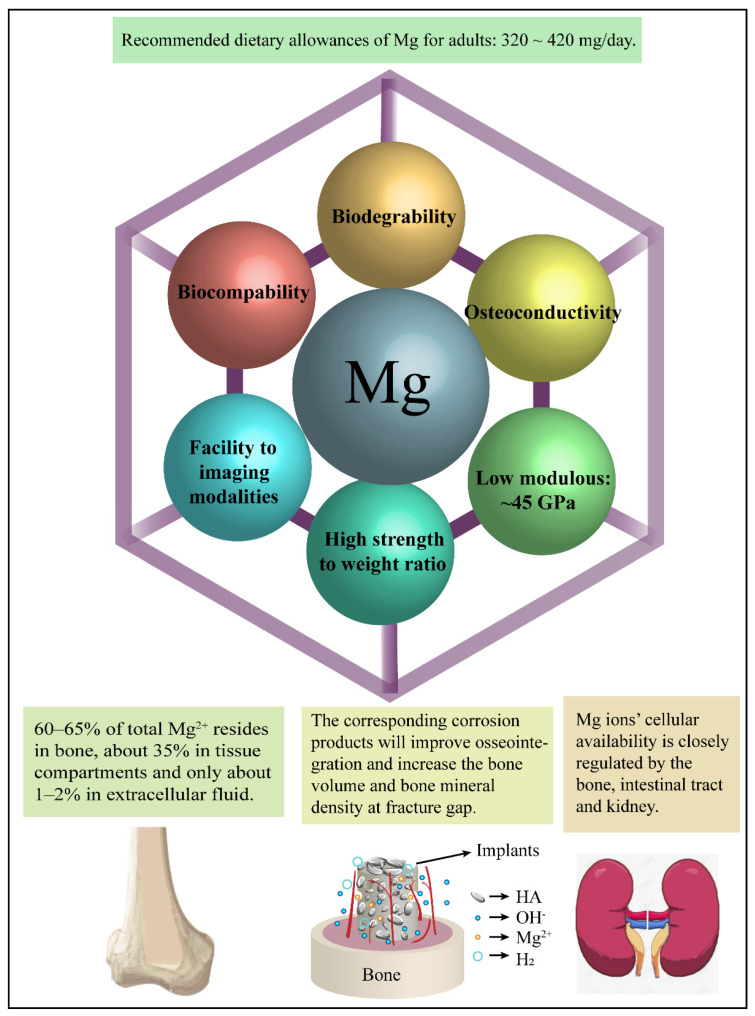
A summary of the advantages of biodegradable Mg-based implants. (1) The balance of magnesium ions can be precisely controlled by the kidney [14,24,42]. (2) Mg and its alloys exhibit an elastic modulus close to that of natural bone [43]. (3) Enhanced alkalinization is not only beneficial for the synthesis and crosslinking of collagen, but it also supports the formation of HA, which provides excellent bioactivity and osteoconductivity [44,45,46].

Great progress has been made in the field of biodegradable Mg alloys in the last decades. However, a review, which covers the latest conceptions of the dynamic corrosion behavior, the domination of the degradation, and the bioactive degradation product, is not comprehensive. Accordingly, this work reviews the corrosion mechanism in Mg alloys, especially the degradation/precipitation reactions and influential factors in the physiological conditions. In addition, the related factor, which affects the application of the biomedical device, is also discussed. A systematic improving technique is proposed for the fabrication of these devices, which could improve their compatibility in clinical applications (Figure 1).

## 2. Corrosion Behavior

Mg and its alloys are generally known for degrading in aqueous environments, primarily through electrochemical reactions (corrosion) that produce Mg hydroxide and hydrogen gas. The specific corrosion reactions are shown below:

Mg ↔ Mg^2+^ + 2e^−^ (anodic partial reaction), (1)

2H_2_O + 2e^−^ ↔ H_2_ + 2OH^−^ (cathodic partial reaction), (2)

Mg + 2H_2_O ↔ Mg^2+^ + H_2_ + 2OH^−^ (overall reaction), (3)

Mg + 2OH^−^ ↔ Mg(OH)_2_ (product formation). (4)

Anodic partial reactions, with a slight decrease in pH [47], typically occur in the corrosive Mg matrix adjacent to cathodic zones (e.g., impurity particles, noble elements enriched regions, second phases) [48,49,50,51]. This Mg matrix is prone to be internal galvanic attacked due to a very negative electrode potential [52,53,54]. Localized alkalinization also occurs at cathodic sites [47,55], with an increased pH value of up to 10–11 [55]. This can approach the passivity region of Mg [53] and lead to the formation of a partially protective outer porous Mg(OH)_2_ film and a dehydrated MgO inner layer [47,56,57,58,59]. Additionally, selective corrosion occurs in Mg-base alloys, and usually, active Mg atoms dissolve preferentially [60,61], contributing to the enrichment of noble elements’ atoms in the inner layer [62,63].

In physiological liquid or serum, the dissolved metallic ions and dissociative calcium (Ca^2+^) ions can react with other anions in the medium and form products such as carbonates, phosphates, chlorides, etc. [59,64,65]. These compounds with low solubility product constant (K_sp_) precipitate as insoluble in the corrosion layer [66,67,68,69], which is beneficial for slowing the corrosion rate [59,70] and stabilizing the local pH [55,56,71]. However, in this process, the hydroxide film can gradually become unstable [72], facilitating the penetration of Cl^−^ ions in this quasi-protective film [73,74,75]. As a result, Mg(OH)_2_ is converted into a soluble substance (MgCl_2_) through the following reaction [32,58,70]:

Mg(OH)_2_ +2Cl^−^ ↔ MgCl_2_ + 2OH^−^. (5)

When the dissolution of hydroxide opens up the inner layer, the hydration effect easily converses the magnesium oxide to laminar hydroxide with a volume twice as original oxide [60,76]. They were expected to slow down the corrosion rate by blocking the anodic areas. On occasion, compressive rupture of the film may also arise, resulting in continual exposure of the inner layer or fresh metal surface [76]. Alternatively, with a low Pilling–Bedworth ratio < 1 (PBR; the ratio of the volume of oxide to the volume of metal) [59,77,78], the exposed discontinuous magnesium oxide promotes the anodic dissolution of Mg alloy, via porosity acting as paths for Mg cations [59,79]. Although supported by supersaturation and subsequent precipitation, a filamentous Mg(OH)_2_ film grows subsequently, the high surface and porous morphologies permit continued access of electrolyte to the inner regions [78,80].

**Figure 2:**
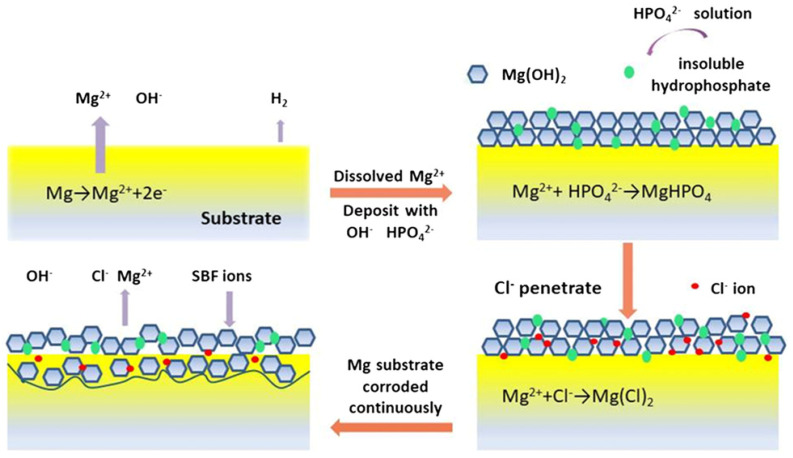
A schematic illustration of corrosion mechanisms in Mg-based alloys in a simulated body fluid (SBF) solution [70].

Therefore active degradation of Mg-based alloys still takes place, even as the corrosion rate decreases gradually with time. This contributes to a continuous cycle of corrosive film formation and decay [55], which consequently increases the coverage of corrosion product layers on metal surfaces [73]. Specific corrosion processes for Mg-based alloys are discussed in the following sections (Figure 2).

### 2.1. Galvanic Corrosion

Galvanic corrosion, also called coupled corrosion, typically occurs during physical or electrical contact in which two types of metals with different electrochemical potentials interact in an ion-conducting fluid medium [81]. It is one of the major obstacles to the application of magnesium components in chemically active environments. Galvanic corrosion in Mg-based alloys can generally be attributed to the poor quality of alloys developed from the enrichment of noble metals [82,83], poor device design, or inadequate assembly practices [84,85,86]. Due to the relatively low solubility of elements in Mg, second phase particles can develop when the alloy is in excess of the solubility limit [87]. Subsequent inter-galvanic effects can strongly influence the initial stages of corrosion in Mg alloys [48,49] (Table 1).

The development of precipitate phases directly affects corrosion rates in Mg-based alloys due to the limited solubility of magnesium [87]. These secondary phases are typically nobler than pure Mg [52,91], contributing to the formation of internal galvanic couples that increase the degradation rate. For instance, Zhou et al., observed an AlCuMg phase that served as a micro-cathode site and was a primary cause of accelerated degradation rates in AZ31-xCu alloys [82]. In addition, Song and Xu [92] reported on precipitated Al_8_Mn_5_ intermetallic phases occurring during heat treatments. Such phases are responsible for the reduced corrosion resistance of AZ31 and can supersede the influence of grain size or crystallographic orientation. However, decreased corrosion rates produced by secondary phases were also reported [93]. When the volume fraction of secondary phases is sufficiently large and a continuous network of precipitated phases is formed, it can retard the corrosion process through a barrier mechanism [94,95]. As such, continuous secondary phases can inhibit the development of corrosion from one grain to another [96]. Furthermore, dual effects in precipitated phases can be affected by distribution morphology when the potential difference is small between the second phases and the Mg matrix. For instance, the polygonal type Mg_2_Si decreases the corrosion current density, while the Chinese script type Mg_2_Si can increase [97,98].

### 2.2. Pitting Corrosion

Localized corrosion occurs preferentially in adjacent Mg matrix [50] since noble secondary phases often act as cathodic regions. This leads to trenching and pitting effects where particles fall out of the substrate, accelerating the rate of corrosion [49,99]. Pitting corrosion is typically aggravated in chloride solutions and exhibits a positive correlation with increasing duration and chloride content [100]. As such, it is often associated with a breakdown in passivity [101]. In addition, the growth of pits tends to spread laterally rather than perpendicularly, covering the entire surface. The inward alkalinization stabilizes the local magnesium hydroxide film and, in turn, decreases the perpendicular pitting corrosion tendency [58].

### 2.3. Stress Corrosion

Magnesium-based implants will possess adequate resistance to failure when applied in the corrosive physiological fluid with a synergetic influence of mechanical loading [102]. The mode of the stress loading can be static or cyclic, which can result in the complications of sudden fracture of implants via the stress corrosion cracking (SCC) and the corrosion fatigue (CF) [103,104]. Generally, SCC is related to anodic dissolution and hydrogen embrittlement [105,106,107,108,109]. It is suggested that the anodic dissolution with intergranular stress corrosion feature can be attributed to the micro-galvanic activity between grain boundary second phases and the adjacent matrix [105,108,110,111,112]. The corresponding corrosion susceptibility can be increased by the electrochemical thermodynamic activity, rupture of the surface layer, and dislocations piling up in grain boundaries or internals [113], accompanied by localized corrosion/pits [108]. The mechanism of the hydrogen embrittlement implies that hydrogen generated during anodic dissolution can be absorbed by the matrix, in turn, inducing hydrogen-assisted cracking [105,113,114]. Transgranular stress corrosion cracking is often attributed to hydrogen interactions [107,114]. However, until now, the exact role of the hydrogen in the SCC of Mg alloys is not fully understood [115]. Recently, research has shown that most parts of the hydrogen were contained in the corrosion products compared to the matrix [116,117].

CF is another type of metallic damage produced by accumulated cyclic loads in high-strain environments. It is caused by the interaction of irreversible cyclic plastic deformations with localized chemical or electrochemical reactions. Meanwhile, it is a critical factor in determining the lifespan of metallic implants. Specifically, devices used for orthopedic or cardiovascular applications must perform higher resistance to corrosion fatigue due to the high-frequency use. Implant lifespans are also heavily affected by the immersion duration [118], the microstructure [119], corrosive phases, and pits [120,121,122]. Kannan et al. suggested the formation of passive films can slow the crack initiation and also the subsequent propagation of fractures [123]. Jafari et al. [124] further proposed that pits often serve as initiation sites for CF cracking, with nucleation and growth influenced by electrochemical conditions and applied stress levels. Therefore, enhanced pitting resistance plays an important role in improving CF resistance in Mg-based alloys [125].

### 2.4. Hydrogen Evolution (HE)

In the magnesium corrosion reactions shown in Equation (3), the evolution of hydrogen results in further structural degradation [59,119]. Immersion in a chloride-containing aqueous solution, along with the initial cathodic activation [126], results in dissolution as the surface progressively darkens in color [61,127]. These corroded regions are cathodically activated and further perpetuate hydrogen evolution [61].

Possible explanations for this process are as follows. First, localized dark regions are likely to accumulate noble elements, thereby enhancing the cathodic reaction rate [72,128]. Second, the disruption of unstable surface films further exposes catalytically active sites [72]. In addition, the unstable and porous Mg(OH)_2_ as the main product induces continuous corrosion on the metallic surface [129,130], and the pores or cracks in MgO film may serve as capillary action [59]. Thereby, recent research has suggested the Mg(OH)_2_ layer plays a dual role in corrosion. It serves as a catalyst for HE but also passivates Mg, which explains the decline of hydrogen evolution reactions (HERs) after prolonged immersion times [131].

### 2.5. Corrosion Behavior in Different Corrosive Environments

#### 2.5.1. Corrosive Electrolytes

Implants are continually subjected to a complex environment containing water, inorganic ions, organic molecules, and proteins. A comprehensive understanding of the interactions between these species and Mg-based alloys is therefore critical to the development of biodegradable metallic implants with optimal biocompatibility and controlled degradation rates.

**Figure 3:**
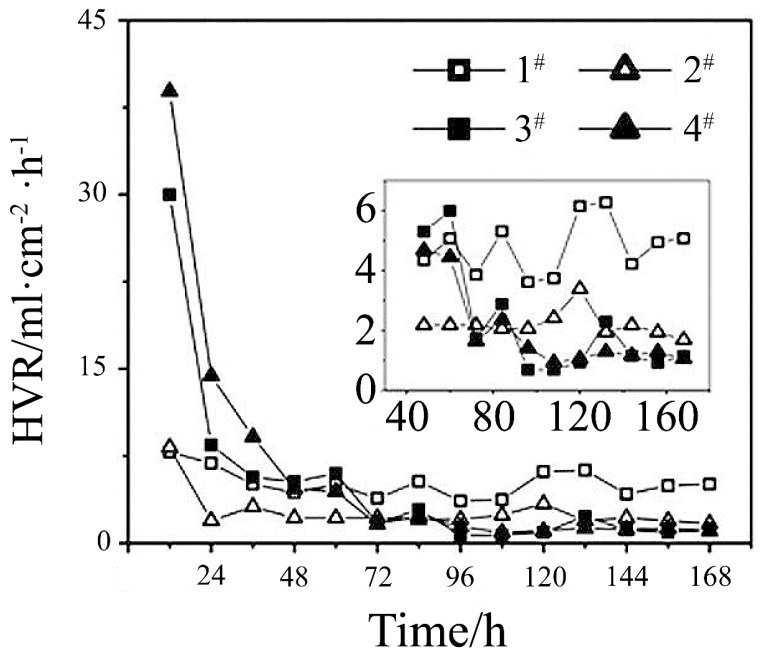
HE rates for an AZ91 alloy as a function of immersion time in the four solutions: 1^#^Cl^−^; 2^#^ Cl^−^ and HPO_4_^2−^; 3^#^Cl^−^, HPO_4_^2−^ and CO_3_^2−^; 4^#^Cl^−^, CO_3_^2−^, SO_4_^2−^, and HPO_4_^2−^ [134].

Corrosion rates are directly influenced by the concentration of aggressive ions. Specifically, Mg-based alloys corrode locally in chloride solutions where the number of pitting sites correlates directly with the concentration of chloride ions [100,132,133]. This effect can be attributed to the involvement of such ions in inhibiting the growth and contributing dissolution of hydroxide, phosphates, and carbonates [75,98]. Contrary, the phosphate anions promote the precipitation of magnesium phosphate, which further decreases the corrosion rate and delays the formation of pitting sites. In addition, the existence of hydrogen carbonate ions stimulates the corrosion of Mg alloys during early immersion stages. However, passivation on the surface can be rapidly induced as well. For instance, the corrosion product MgCO_3_ precipitates on the surface and subsequently suppresses pitting. Protective layers can thereby form more rapidly and effectively with higher concentrations of hydrogen carbonate ions, as evidenced in SBF solutions with HCO_3_^−^ of 27 mmol/L [69]. However, sulfate ions induce the stimulated dissolution of magnesium, as evidenced in solutions with different anions, including 1^#^Cl^−^, 2^#^Cl^−^ and HPO_4_^2−^, 3^#^Cl^−^, HPO_4_^2−^ and CO_3_^2−^, 4^#^Cl^−^, CO_3_^2−^, SO_4_^2−^ and HPO_4_^2−^ [134] (Figure 3).

**Figure 4:**
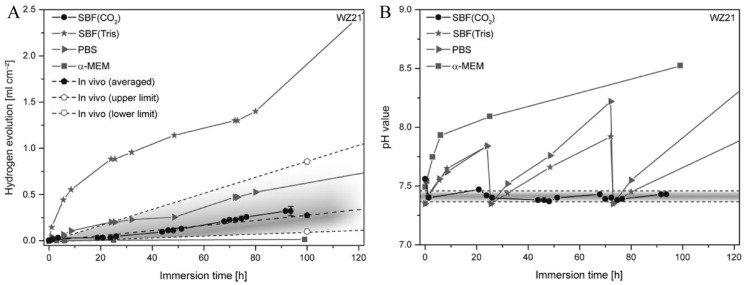
In vitro degradation performance for an SBF-CO_2_ WZ21 alloy, which is in agreement with the average in vivo degradation rate based on µCT (computed tomography) data. The pH values remained mostly constant. (**A**) H2 evolution over time of WZ21 immersed in different physiological media; (**B**) Corresponding pH values evolution in the testing solutions over immersion time [140].

Physiological pH is essentially maintained at 7.4 in vivo due to an equilibrium between bicarbonates, carbonic acid, and carbon dioxide. In plasma, buffered systems include plasma proteins and hemoglobin [135,136]. In contrast, zwitterionic buffers, such as 4-(2-hydroxyethyl)-1-piperazineethanesulfonic acid (HEPES) or tris (hydroxymethyl) aminomethane (Tris) are commonly used to maintain a constant pH value for in vitro immersion studies, without the effects of ionic interactions [137,138]. Corrosion behavior in Mg alloys differs in various buffer systems. For example, previous studies reported an increased corrosion rate for Mg in HEPES buffered solutions, compared with those of CO_2_ buffered solutions (i.e., Earle’s balanced salt solution, minimum essential medium (MEM), or MEM-containing bovine serum albumin) [139]. Analogously, the degradation of Mg–2Y–1Zn–0.25Ca–0.15Mn (WZ21) alloys in SBF-Tris is approximately five times faster than that of SBF-CO_2_. However, in HEPES (100 mmol/L), the rate of degradation increases by approximately 60 times [140]. This accelerated corrosion rate contributes to a lower-density protective layer, which results in the increased diffusion of aggressive ions [137]. The use of Tris-HCl in SBF lowers the corrosion potential of pure magnesium slightly but accelerates the early degradation of pure magnesium by teens times. Tris-HCl also increases pitting corrosion sensitivity in magnesium [141] (Figure 4).

Proteins are generally adsorbed on surfaces through complex processes (e.g., Van der Waals forces, hydrophobic, electrostatic interactions, and hydrogen bonding). Conflicting reports of both increasing and decreasing corrosion rates with protein addition were published [137]. These increased corrosion rates were explained by protein chelation of Mg^2+^ [75], which increases the dissolution of Mg(OH)_2_ film on the surface and decreases the precipitation of other corrosion products, such as phosphates and carbonates. These decreased corrosion rates can be attributed to the adsorption of protein in corrosion products [142], as proteins are negatively charged when pH values exceed 7 [143,144]. This diffusion of ions from interfaces can be successfully prevented by adsorption. Consequently, an intermediate passive layer with amino acid protein debris could be produced to form a buffer between the environment and the material [145].

#### 2.5.2. In Vitro and In Vivo Environments

The influence of cells on corrosion behavior in Mg-based biomaterials is normally discussed from two competing perspectives. First, corrosion can be alleviated using a physical barrier produced by the cell body and extracellular matrix, consequently hindering the encroachment of corrosive ions. Seuss et al., cultured a human cervical cancer cell line (HeLa) on the surface of an AZ91D alloy and used electrochemical impedance spectroscopy to observe the increased impedance caused by adherent cell layers, which slowed electrochemical reactions during corrosion [146]. Second, cells can disturb microenvironments between metal and medium interface during metabolization (e.g., pH alteration, ion transport, and the secretion of inorganic or organic molecules), which decreases the corrosion resistance of Mg-based alloys [29]. Kannan cultured mouse fibroblast L929 cells on the surface of an Mg–Ca alloy and found that polarization resistance decreased with increasing culture periods. Pitting also occurred in the vicinity of the L929 cell bodies, which was attributed to a reduction in pH caused by cell metabolism [147]. Zhang et al. [148] cultured RAW264.7 macrophages on an Mg–Nd–Zn–Zr alloy. The reactive oxygen species (ROS) produced and secreted by macrophages diffused through the corrosion product layer and presumably enhanced the formation of cavities through a reaction with the Mg-based alloy matrix.

In vivo degradation rates depend strongly on the location of an implant within the body [15,149]. Previous studies suggested that the degradation rate accelerates in surrounding tissues with higher degrees of vascularization, as the enhanced exchange of bodily fluid promotes the degradation of materials [31]. For instance, the medullary cavity (a region with higher vascularization) exhibits higher rates of degradation than cortical bone (with lower vascularization) [33,150]. A similar effect can be observed in liver tissues and the rectus abdominis muscle [34]. Load bearing conditions also play an important role in degradation rates due to the possibility of stress corrosion and corrosion fatigue. Willbold et al. observed that magnesium screws composed of an AZ31 alloy exhibited a higher corrosion rate in bone than in overlying muscle or connective tissue [151]. In addition, crevice-enhanced corrosion can occur in bone–bone plate interfaces, thereby increasing corrosion rates through absorbing chloride ions into the crevice [152].

## 3. Influential Factors for Corrosion Behavior in Magnesium

### 3.1. Alloy Composition

The existence of two competing mechanisms via altering composition leads to a complicated corrosion process in Mg-based alloys [96,153]. Micro-galvanic corrosion can be induced by the formation of intermetallic phases, consequently increasing the degradation rate [82,96,99,154]. In contrast, the formation of a more protective surface layer can effectively protect the substrate, which decreases the corrosion rate [73,74,155,156]. The corrosion behavior of Mg-based alloys is thus directly affected by the content of elements and the formation of corrosion products [157]. Additional details for processes involving various elements are provided in the following sections.

#### 3.1.1. Non-Rare Earth (RE) Elements

Calcium (Ca) is a major component in human bone and is essential in cellular chemical signaling [158]. Prior studies showed that existing forms of Ca can heavily influence the corrosion rate of the Mg matrix [159]. Corrosion resistance remains high provided Ca is available in the form of dissolved atoms in a solid solution. At the eutectic temperature of 517 °C, Ca has a maximum solubility of 1.34 wt% in Mg, and another constituent *β* phase, Mg_2_Ca, forms with Ca content over Mg solid solubility [160]. Compared with pure Mg-based alloys, Mg_2_Ca is highly reactive [161]. It exhibits a pretty negative corrosion potential and sustains a dissolution rate approximately one order of magnitude higher than pure Mg. Therefore, the increasing content of the Mg_2_Ca phase results in a higher corrosion rate in Mg-based alloys [32,162]. The use of potentiometric micro-probes has revealed several noteworthy effects. For example, in the presence of HPO_4_^2^^−^ and HCO_3_^−^, calcium ions play a major role in stabilizing local pH and decreasing degradation rates [163], which contributes to the enhanced formation of calcium phosphate on a surface [25]. Previous studies have indicated that improved corrosion resistance can be achieved in multi-component Mg-based alloys with a reduced addition of Ca (≤1 wt%), which results in stabilization of the corrosion layer and increased corrosion resistance [96,159,164,165].

Zinc (Zn) is the second most abundant transition metal in the human body and is an essential element for biological functions such as nucleic acid metabolism, signal transduction, apoptosis regulation, gene expression, modulation of brain excitability, endocrine regulation, and interactions with a wide range of organic ligands [166]. The limited addition of Zn in Mg-based alloys can also cause a reduction in the degradation rate [167]. As reported by Bakhsheshi-Rad, the corrosion rate for Mg–Zn binary alloys significantly decreased with increasing Zn content (up to 4 wt%) due to the formation of a more compact Zn containing inner oxide layer [168]. However, the max solubility for Zn in Mg is 6.2 wt% at eutectic temperature (Table 1), which becomes lower at room temperature. The further increases in Zn decreased corrosion resistance due to increased precipitation phases at grain boundaries and enhanced galvanic effects [169]. Similar processes were observed in Mg–Zn–Zr systems [153].

Silver (Ag) exhibits an outstanding antibacterial reactivity in a variety of chemical states [170,171]. The presence of Ag in Mg–Ag binary alloys was identified in the forms of Mg_3_Ag and Mg_54_Ag_17_, which provide higher corrosion resistance than the α-Mg matrix. They can also serve as highly active micro-cathodes, which form numerous micro-corrosion cells on a material surface, promoting electrochemical reactions [172]. An increased electrochemical corrosion rate was also observed in Mg–9Al–Zn–xAg (x = 0.12, 0.5 in wt%) alloy with relatively high Ag content (0.5 wt%), owing to enhanced cathodic kinetics sustained in the Mg_4_Ag phase [173]. Nevertheless, the development of pitting corrosion could be restricted by the formation of AgCl in corrosion products and a decreased grain size with Ag addition [65].

Strontium (Sr) is a necessary component of human bone and has been used to enhance mineral density in osteoporosis treatments. The solid solubility of Sr in Mg is 0.11 wt%, making it easy to segregate. The mean grain size in Mg-based alloys can thus be refined with small additions of Sr (<1 wt%) [174,175,176], contributing to a decreased corrosion rate. However, excess Sr produces the opposite effect, increasing the corrosion rate at concentrations above 1 wt% [177].

Tin (Sn) has a high liquid solubility in molten Mg (14.85 wt% at 561 °C) and low solid solubility (0.17 wt%) at room temperature [178,179]. When Sn is dissolved in the magnesium matrix using heat treatments, the porous outer layer on the surface film becomes more compact, and Sn enriching in the inner layer can significantly lower the HE rate [180]. Increasing Sn content can also decrease the defect density of surface films. As a result, the adsorption of chloride ions is inhibited, and the corrosion resistance is correspondingly enhanced [181]. However, further addition of Sn (up to 2 wt%) brings out a great cathodic effect with excessive Mg_2_Sn phases, which can achieve accelerated degradation [179].

Although manganese (Mn) has limited solid solubility in Mg (~0.95 at% at 650 °C), it improves corrosion resistance through decreasing free Fe contain and formation of relative innocuous intermetallic compounds (e.g., Al*_x_*(Mn,Fe)*_y_*) in Mg–Al alloys [182,183,184]. Mn has also displayed moderating effects on Fe and cathodic kinetics in Al-free systems [185,186]. On the one hand, the cathodic activation behavior of pure Mg is inhibited in Mg–Mn alloys via forming Mn(Fe) phases, which not only creates a buffer zone with a weakened micro-galvanic effect but also inhibits the redeposition of the Fe element to the magnesium surface [187,188,189]. On the other hand, metallic Mn has a standard electrode potential closed to the Mg (Mn: −1.185, Mg: −2.372, Fe: −0.447); thereby, the cathodic activation induced by the Mn element is relatively weak, and consequently, HER is inhibited via the formation of dense Mn-rich corrosion layer [187,190]. Additionally, the element of Mn was reported to contribute to the formation of the transition layer at the interfaces of Fe/Mg diffusion couples at 700 °C, which effectively inhibits the diffusion of Fe atoms in the Mg–2Mn melt [191]. However, Yang et al. observed that the evolution of H_2_ increased in Mg–3Zn–xMn alloys when the content of Mn was higher than 0.5 wt% [192].

Zirconium (Zr) acts as a Fe scavenger and a grain refiner in Mg-based metals [193,194]. In Mg–Zr alloys, the degradation rate depends primarily on Zr concentrations and distributions. A high Zr content in solid solutions (~0.5 wt%) can activate anodic reactions, thereby accelerating the general corrosion of the alloy matrix and increasing the long-term corrosion rate [186,195,196,197]. In addition, barrier effects in the second phase can be weakened as superfluous Zr particles congregate at grain boundaries [198]. However, in several ternary (and higher-order) alloys, the addition of Zr can result in the stabilization of solid magnesium solutions, inactivating the material during anodic dissolution and reducing cathodic hydrogen evolution [50,193,199].

#### 3.1.2. Rare Earth (RE) Elements

Rare earth elements (REEs) often display ‘scavenger’ tendencies [200,201], leading to the formation of more stable oxide films. This can decrease the occurrence of micro-galvanic corrosion between Mg and impurity elements, such as alloying with La, Ce, Er, Ho, etc. [96,202,203,204]. While light REEs are toxic [205,206,207,208,209], heavy REEs are often used for biomedical materials owing to their high solubility and resistance to forming intermetallic phases. The degradation rates of binary Mg–Y, Mg–Dy, and Mg–Gd alloys depend on the concentration of each alloying element. Optimal amounts for Y, Dy, and Gd are 2 wt%, 10 wt%, and 15 wt%, respectively. In Mg–Y binary alloys, intermetallic compounds can accelerate micro-galvanic corrosion. In contrast, oxide films containing Y can enhance surface protection [210,211,212,213]. The presence of Dy in Mg–Dy alloys can lead to the formation of protective layers. The corresponding oxide product (Dy_2_O_3_) can effectively increase degradation resistance [214]. In addition, the Mg_2_Dy phase formed at grain boundaries during non-equilibrium cooling is able to reduce galvanic effects [215], achieving enhanced corrosion resistance. Gadolinium (Gd) has a higher affinity to oxygen compared with Mg. As such, the corresponding corrosion products (Gd_2_O_3_ and MgGd_2_O_4_) exist in the form of particles on the surface and act as a diffusion barrier to prevent further oxidation.

### 3.2. Organizational Structure

#### 3.2.1. Crystal Structure

In crystalline Mg alloys, grain size plays a significant role in the formation of oxide films [216,217]. Ralston et al., showed that the corrosion rate of metals is proportional to the reciprocal square root of the grain size [218]. In addition, thin inner-magnesium oxides can form on surface corrosion layers. Grain refinement increases the volume fraction at grain boundaries and consequently decreases the mismatch between crystal lattices in the Mg matrix and oxide layers, which creates a more adhesive interface [219]. As such, denser grain boundaries result in higher corrosion resistance [161,218,220]. In addition, grain-refined Mg surfaces exhibit accelerated passivation kinetics in chloride-containing electrolytes, which can effectively withstand breakdowns through the rapid formation of magnesium oxide films [221,222]. Huang et al., demonstrated that grain refinement could also delay the propagation of stress corrosion cracks, which are hindered at grain boundaries [220].

**Figure 5:**
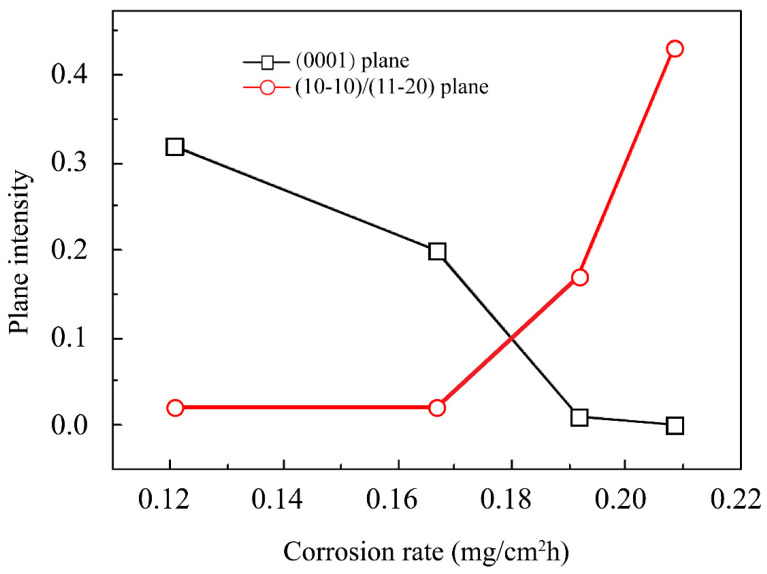
Correlation between the corrosion rate and intensity planes in an AZ31 Mg alloy in 3.5 wt% NaCl [227].

Crystallographic orientation is an important microstructural characteristic of metals. Different crystallographic planes exhibit varying surface energy levels, leading to various corrosion behaviors in a given environment [223,224]. Closely packed planes exhibit higher binding energies due to increased atomic coordination. This results in lower surface energy with weakened anodic dissolution and adsorption for water or protons [225]. The surface energies in Mg (0 0 0 1), (1 0 $\overset{¯}{1}$ 0), and (1 1 $\overset{¯}{2}$ 0) (here $\overset{¯}{x}$ represents negative *x* value) planes are 1.808, 1.868, and 2.156 eV/nm^2^, respectively [226]. These values can be converted to 1.54 × 10^4^, 3.04 × 10^4^, and 2.99 × 10^4^ J/mol, respectively [227]. In comparison, other crystallographic planes exhibit much larger electrochemical degradation rates than that of the base plane (0 0 01) [92,228]. This indicates that exposed surfaces containing primarily (0001) planes exhibit higher corrosion resistance compared to surfaces composed primarily of (1 0 $\overset{¯}{1}$ 0) or (1 1 $\overset{¯}{2}$ 0) planes [225]. Although orientation was recommended as a first-order effect for Mg-based alloy degradation [225], it could be less significant compared with the effects of precipitation and varying grain size [49,92,229] (Figure 5).

#### 3.2.2. Amorphous Structures

Compared with crystals, amorphous Mg-based alloys offer increased solubility for alloying elements and exhibit a homogenous structure without defects such as grain boundaries, dislocations, or precipitates, thus providing excellent corrosion resistance. Known amorphous alloy systems include Mg–Cu–Y [230,231], Mg–Zn–Ca [232,233,234], and other Mg–TM–RE [235,236] alloys (where TM refers to a transition metal). These amorphous alloys process relatively positive corrosion potentials when compared with other crystalline Mg-based alloys in quiescent 0.1 M NaCl solutions, which is evidence of a lower corrosion tendency [52]. Zberg et al., developed a successful Zn-rich MgZn_35__−*x*_Ca_5_ (*x* = 0–7) system with the same biocompatibility as crystalline Mg implants, significantly reducing the evolution of hydrogen [237].

## 4. A Methodology for Improving Corrosion Resistance

### 4.1. Composition Design

**Figure 6:**
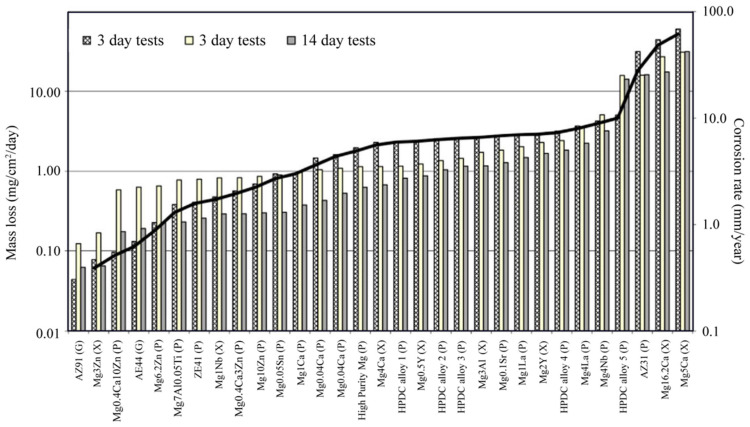
Experimentally determined degradation rates, which differ from Mg-based alloy composition at 37 °C in MEM [238].

The primary objective of composition design is to improve corrosion resistance by optimizing the size and number density of intermetallic particles and scavenging impurity transition elements, to enhance the stability of surface films. Kirkland et al., found the degradation of Mg alloys can be slowed by tailoring compositions, lowering the mass loss rate in SBF by three orders of magnitude [238] (Figure 6).

Recent studies suggested that hydrogen evolution can be prevented when alloying elements (Ca, Sc, Ti, Y, and Zr) bind with adsorbed hydrogen. Additionally, hydrogen absorption can also be inhibited by the addition of Al, As, Cd, Ga, Ge, In, Si, Sn, Sb, and Zn [239]. For example, Liu et al., demonstrated that micro-alloying additions (Bi, Ge, Pb, Sb, and Sn) could decrease cathodic kinetics in binary Mg alloys.

Given the dual effect of Mg hydroxides in HER, the structural optimization of corrosion films should be investigated further. Previous research suggested that the presence of Al in films and Zn at metal-film interfaces can reduce the degradation rate of Mg alloys [127]. These elements are less active and show PBR ranging from 1 to 2, which induces a more dense inner oxide layer [77]. In addition, the proportion of quasi-passivate Mg corrosion products in the surface layer can also be reduced when the K_sp_ of the alloying element corrosion products is lower than that of Mg counterparts [67,68] (Table 2).

### 4.2. Heat Treatment

Solution treatment, also called T4 treatment, is the most frequently used heating technique for improving corrosion resistance in Mg-based alloys [242]. The process involves heating an alloy and maintaining the sample at a suitable temperature for a sufficient time period in order to promote the desired constituent formation into solid solutions. This is followed by rapid cooling to fix the constituent in the solid solution, sufficiently decreasing the precipitates and redistributing elements in the Mg matrix [192]. This process can further reduce galvanic corrosion [243,244]. However, T4 treatment is not suitable when the second phase acts as a corrosion barrier [212,245]. 

**Figure 7:**
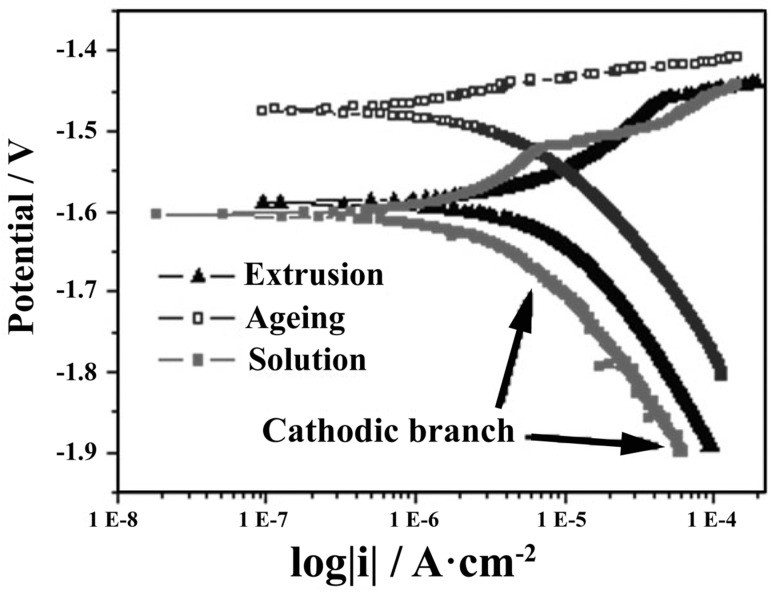
Polarization curves for Mg–6Zn in various states. The T4 treated samples exhibited the lowest corrosion current density, followed by T6 and the extruded samples [169].

T6 treatment, also known as precipitation heat treatment, involves the use of a solution treatment at high temperatures and subsequent artificial aging [242]. T6 produces a slightly lower corrosion resistance than T4, as the precipitates enhance galvanic couples [169,246]. Given the dual influence of secondary phases in corrosion, such precipitates may also provide further corrosion resistance. Li et al., investigated the effects of heat treatments on microstructure and corrosion resistance in Mg–5Zn–1Mn alloy tubes [246]. Although the resistance of T6-treated ZM51 alloys was lower than that of T4-treated alloys, it was superior to that of extruded samples. Wang et al., demonstrated the ZYbK620-T6 alloy also exhibits a favorable corrosion resistance, which may be attributed to the corrosion barrier effect in more uniform and compact passive films induced by dispersed nanoscale precipitates [247] (Figure 7).

### 4.3. Severe Plastic Deformation (SPD)

In recent years, SPD technology has become increasingly popular for processing Mg alloys and isolating fine- and ultra-fine-grained (UFG) materials. This includes hot extrusion [219], equal channel angular pressing (ECAP) [101], isothermal extrusion [248], and high-pressure torsion (HPT) [249]. SPD also induces complex microstructure features, thereby modifying the size, distribution, and morphology of grains and precipitates [49,250]. Zhang et al., reported this process increased the corrosion rate of AZ31B alloys by more than 30% while decreasing the extrusion ratio (ratio of the cross-sectional area of the pressure cylinder cavity to the total cross-sectional area of the extruded product) from 44.4 to 11 [251]. Lin et al., observed that extruded Mg–Zn–Zr alloys with fine grains displayed favorable degradation (with corrosion rate of 5.0 mmy^−1^), cytocompatibility, and hemocompatibility [248]. Similarly, pure magnesium processed by HPT exhibits an improved corrosion resistance [249]. However, SPD includes inherent limitations due to the inevitably of defects such as dislocations, grain boundaries, unfavorable orientations (e.g., exposed (1 0 $\overset{¯}{1}$ 0) basal planes primarily to the electrolyte), segregation of alloying additives, and residual stress [49,252]. Ultimately, these changes to the surface affect the electrochemical behavior and consequently increase corrosion susceptibility [49,101,249,253].

### 4.4. Surface Modifications

Surface modification is an effective approach to improving corrosion resistance and surface bioactivity [254,255,256]. It can be used to improve surface integrity, fabricate physical barriers, and isolate the underlying Mg matrix from corrosive media, thereby maintaining the mechanical integrity of Mg-based alloys before healing is completed.

#### 4.4.1. Surface Machining Processes

Cryogenic machining is a hybrid manufacturing process that focuses on modifying the surface integrity of components, contributing to the improved corrosion resistance of Mg alloys [257,258], owing to the generation of a grain refinement layer with compressive residual stresses [259]. It was reported that the pitting resistance could be improved by cryogenic machining due to the presence of a grain refinement layer, larger compressive residual stresses, and a faster formation of passivating surface oxides [260]. Cryogenic cooling in combination with ultrasonic vibration-assisted turning has been explored in recent years, which not only improves the surface integrity, texture, and wettability but also modifies a complex and isotropic texture for the following coating process [261]. Additionally, the burnishing process is another kind of surface process for reducing surface roughness, which is able to increase the hardness and introduce the beneficial compressive residual stresses near the surface [262]. Reduction in the grain size can be apparent during burnishing applied with the liquid nitrogen [263]. It is also suggested that strong basal texture evolution occurs on the burnished surface, suggesting the basal plane could be much larger by cryogenic burnishing [264]. After the burnishing process, the improvement of the corrosion resistance occurs via a modified surface roughness, the grain size, and the crystal orientation [265].

#### 4.4.2. Ion Implantation

Ion implantation is regarded as an important surface modification technique to obtain a non-abrupt interface with a combination of enhanced physical and chemical properties. When the plasma-treated Mg alloy is immersed in a physiological liquid, the surface-ionized metals with positive charges will interact with the negatively charged microbial cell membranes, producing good antimicrobial properties [266]. Meanwhile, the oxygen vacancies produced in the plasma treatment can result in ROS generation to enhance the antibacterial properties [267]. The decelerated and accelerated degradation effects were reported previously. Yang Liu et al. divided implanted ions into two groups: type I refers to the ions prone to remain in a metallic state, serving as severe cathodic sites and inhibiting the formation of the dense oxidized layer, such as Zn, Ag, and Fe. On the contrary, type II indicates implanted elements are likely to form compact oxidized layers, such as Al, Ti, Zr, and Y. It was suggested that dual ion implantation of the metallic ion and the oxygen promote corrosion resistance. In addition, the post-oxidation treatment combined with ion implantation can be another solution [190,268].

#### 4.4.3. Coating Technology

The oxidative form of some nontoxic or bioactive metal elements can be used to modify the degradation properties of Mg-based alloys in the deposition layer, known as metal oxide implantation [269,270]. For example, the deposition of a 100-nm-thick ZrO_2_ coating on an AZ31 Mg alloy was shown to reduce corrosion rates and hydrogen evolution, which is a result of lower SCC susceptibility for the ceramic coating [271]. Besides, no toxic compact metal oxides with bioactive degradation products are recommended, as they can not only act as a physical barrier but also result in an increase in cell growth and a reduction in cell death [272]. In addition, physical vapor deposition of an Ag–ZnO coating on the substrate can considerably reduce corrosion rates and improve antimicrobial properties [171].

Hydroxyapatite is a primary component of natural bone with excellent bioactivity and osteoconductivity [44,45]. HA coatings on Mg alloys provide superior mechanical stability and stimulate bone growth in vitro, which improves biological response (e.g., cell attachment, proliferation, and differentiation) [273]. In addition, the bonding between bone and implants can be enhanced significantly [274], owing to the retarded degradation of the implant, which has been proven by clinical, radiological, histological, and hematological evaluations [27].

Biocompatible polymer coatings such as chitosan [275,276], polylactic acid (PLA) [277,278], and polycaprolactone (PCL) [278,279] can effectively decrease the degradation rates of implanted materials and facilitate the healing of bone [279,280]. This also supports improved biological performance without affecting the flexibility of Mg alloys by increasing chemical compatibility with surrounding tissue and promoting the growth of new bone [279,280]. However, adhesion between polymeric coatings and Mg-based alloys is poor, which can cause problematic detachment during use [278]. Many papers have covered this in recent years. It was demonstrated the adhesion strength could be improved by some pretreatments, such as cryogenic cooling in combination with ultrasonic vibration-assisted turning, alkaline-treatment, and ceria embedded microarc oxidation process [261,281,282].

Besides the corrosion resistance and the biocompatibility, the bio-functional property has raised a great deal of concern in recent years. By means of introducing specific ions, ceramics, compounds, proteins, etc., or loading commercial drugs on surface coatings, the design of complex and composite coatings has been stimulated [283,284,285,286,287]. For instance, Fe_3_O_4_–HA–chitosan coatings can be prepared by electrophoretic deposition, which displays a superior antimicrobial efficacy than HA coating since Fe^2+^ can induce the generation of H_2_O_2_, damaging the DNA and proteins in bacteria [288]. Moreover, electrospun PCL fibers loaded with coumarin and ZnO on the AZ31 Mg alloy perform a positive inflammatory response and an exerted effect on the macrophage–osteoclast differentiation process [289]. Recently, the etidronate and 3-aminopropyl trimethoxysilane was successfully explored, where the etidronate was known as bisphosphonates applied in the treatment of bone loss and osteoporotic fractures [290]. Furthermore, some studies demonstrated that graphene oxide–chitosan coating with the incorporation of heparin and the bone morphogenetic protein could synergistically improve corrosion resistance, anticoagulation, and osteogenesis [291].

## 5. The Positive Biological Effects of Mg Alloys

### 5.1. Magnesium Ions

Recent research focused on the mechanism of induced osteogenesis with Mg^2+^ ions, suggesting that Mg alloys improve bone formation via the CGRP-mediated cross-talk pathway. The activated CGRP receptor can trigger phosphorylation of CREB1 via cAMP and promote the expression of genes promoting osteogenic differentiation [292]. In addition, Mg^2+^ ion suppresses osteoclastogenesis in dependence on the concentration. Additionally, a high Mg^2+^ concentration in culture media exhibits a significant inhibitory effect on osteoclast formation because of its inhibiting effects on osteoclastogenesis-related gene (NFATc1) expression [293]. In addition, Mg^2+^ simultaneously improves the phosphorylation of ERK (enhanced c-fos levels) and GSK3β (enhanced β-catenin levels). It was also proposed that magnesium can promote distraction osteogenesis consolidation via regulating Ptch protein activating Hedgehog-alternative Wnt signaling [294]. As such, dissolved Mg^2+^ promotes the proliferation and differentiation of osteoblasts, producing positive biological effects in stimulating bone formation [295]. Meanwhile, in terms of inhibiting cancer aspects, a high concentration of Mg^2+^ (≥30 mmol/L) has shown an inhibiting effect on the proliferation of gallbladder cancer cells and induces apoptosis [296].

### 5.2. Hydroxyl Ions

Due to the presence of hydroxyl ions, enhanced alkalinization is not only beneficial for the synthesis and crosslinking of collagen but also supports the formation of hydroxyapatite, which effectively promotes bone growth and repair in vivo [297]. Furthermore, related alkaline pH is sufficient to induce antibacterial effects, promoting a modest reduction in bacterial activity in vivo [298,299,300]. Hydroxyl ions were also reported to inhibit the proliferation or induce apoptosis of gallbladder cancer cells when the medium pH was greater than or equal to 7.8 [296].

### 5.3. Corrosion Layers

**Figure 8:**
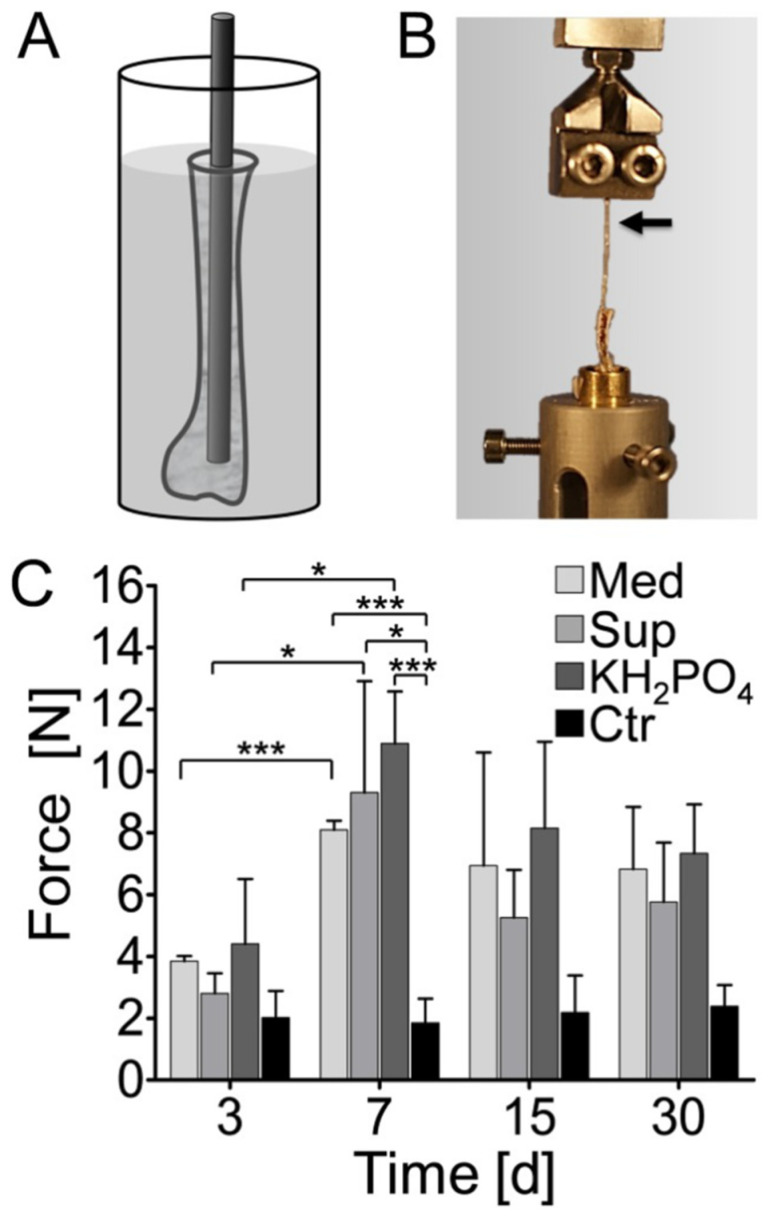
(**A**) The Mg pins were inserted into the medullary cavity of sterile murine tibia bones and incu-bated in different corrosion solutions at 37 °C and 5% CO2; (**B**) After incubation, the pull out force between the bone and the the magnesium pin were measured; (**C**) Such Mg pins exhibited an increased pull-out resistance in the absence of live cells. Abbreviations are as follows: Med, group incubated in freshly prepared cell culture medium; Sup, group incubated in cell culture supernatant; KH_2_PO_4_, group incubated in 100 mM KH_2_PO_4_ buffer; Ctr, group incubated in fresh cell culture medium [46]. A *p* value < 0.05 was considered statistically significant, where 0.01 ≤ *p* < 0.05 marked by *, 0.0001 ≤ *p* < 0.001 marked by ***.

Undissolved surface hydroxide layers in Mg-based alloys provide favorable sites for HA nucleation [301]. This nucleation activity can be accelerated by improving the supersaturation of hydroxyapatite in solutions with an accompanying increase in pH value [32,302]. This is evidenced by the superior osteoconductivity of the phosphate layer, which maintains tight contact between implants and bone [15]. Specifically, the accumulation of corrosion products gradually exerts pressure on adjacent bones, stimulating the fortification and growth of bone according to Wolff’s law [46] (Figure 8).

## 6. Outlook

Increasing the corrosion resistance of degradable materials is critical for developing improved Mg-based alloys for biomedical applications. Reduction in the degradation rate of magnesium not only creates a relatively stable interface for cell adhesion and growth but also declines the release of corrosion products that can compromise cytotoxicity. The specific implants (e.g., cardiovascular stents and orthopedic devices) require a minimized degradation rate and high mechanical support. In terms of imparting natural corrosion resistance to alloys, composition design is the most fundamental method. On the one hand, reducing the galvanic effects is critical; for instance, select elements with low cytotoxicity and high solubility for alloying. Besides, the potential difference between the main alloying elements and magnesium is better minimized [62]. In addition, non-equilibrium techniques and solution treatment are promising to inhibit precipitates [303]. Moreover, REEs, Mn, and Zr can be involved in scavenging impurities.

On the other hand, increasing the stability and density of corrosion products layers is also important, and the reasonable method is to reduce the fraction of Mg corrosion products. First, the element with lower K_sp_ of corrosion products than Mg counterparts (as shown in Table 2) should raise more concern, as they will induce a more stable and insoluble film on the surface layer. Second, replacing Mg with other elements with PBR ranging from 1 to 2 can potentially induce the formation of a more compact oxide’s inner layer [59,77]. Third, elements with decreasing cathodic kinetics effect are also promising to serve as alloying candidates because it suppresses the film disruption caused by increasing volume and pressure of hydrogen [59]. Lastly, comprehensive utilization and further optimization of composition design, heat treatments, and various surface modification techniques are needed to overcome magnesium alloys’ poor resistance [261,304].

By considering the complexity of in vivo environments, comprehensive research on synthetic body fluids is necessary to achieve a sufficient understanding of the corrosion mechanisms present in Mg-based alloys. The most essential task is identifying critical parameters and factors (e.g., aggressive ions, buffered systems, proteins, and cells) that strongly affect corrosion behavior, providing more practical information for simulating in vivo measurements [134,141,305]. Given the recent progress of first-principles methods, it is promising to describe the thermodynamic stability of the formation of complex corrosion products, which is able to reevaluate the electrochemical phase diagrams in physiological fluid [305]. It will bring a new sight for the composition design of Mg alloys, further contributing to the exploration of Mg-based biomaterials with enhanced corrosion resistance, desired corrosion products, and improved clinical applications.

## 7. Conclusions

In this review, we focused on the complicated, influential factors behind corrosion behaviors and possible routes to optimize the intrinsic effect. The main conclusions are as follows:

(1) An attempt was made to describe the corrosion behavior of Mg alloys based on the dynamic degradation/precipitation model, which is applicable for Mg alloys with Mg hydroxides as one of the main passivation layers in physiological conditions;

(2) Generally, the inherent low corrosion resistance of Mg alloys can be attributed to a dramatic negative electrode potential and quasi-passivate corrosion product layers. The latter process a multi-layered structure with Mg(OH)_2_ film function as a medium layer, which is less compact/insoluble, and serves as a catalyst for HE. It is important to reduce the fraction of Mg corrosion products through alloying passivating elements into a matrix, conducting ions/oxide implantation on the metallic surface;

(3) The effective factors behind Mg corrosion behavior can be classified into intrinsic and extrinsic factors. With regard to the intrinsic influence, it is significant for the determination of the corrosion behavior in the given condition, which implies the basic feature, e.g., the composition, the microstructure, and the surface condition. The composition design combined with non-equilibrium techniques (heat treatment, SPD, and surface treatments) is critical for improving degradation resistance and pitting susceptibility. The comprehensive utilization of modification methods is promising to meet the need of the degradation resistance in vivo.

(4) In terms of biodegradation, the application of Mg alloys is promising and challenging due to their positive biological effects. The degradation rate is still needed to be modified by means of innovative fabrication techniques and surface treatments in order to fit the tissue growth.

## Figures and Tables

**Table 1:** Solubility data for binary Mg-based alloys [88,89,90].

Element.	Atomic%	Weight%	System	Ref.
Lithium	17	5.5	Eutectic	[88,89]
Aluminum	11.8	12.7	Eutectic	[88,89]
Silicon	~0.00	~0.00	Eutectic	[90]
Calcium	0.82	1.34	Eutectic	[89,90]
Titanium	0.1	0.2	Peritectic	[88,89]
Manganese	1	2.2	Peritectic	[88,89]
Iron	~0.00	~0.00	Eutectic	[90]
Copper	0.02	0.04	Eutectic	[90]
Zinc	2.4	6.2	Eutectic	[88,89]
Strontium	0.03	0.11	Eutectic	[90]
Zirconium	1	3.8	Peritectic	[88,89]
Silver	3.8	15	Eutectic	[88,89]
Cadmium	100	100	Complete SS	[88,89]
Indium	19.4	53.2	Peritectic	[90]
Tin	3.35	14.48	Eutectic	[88,89]
Gold	0.1	0.8	Eutectic	[88,89]
Yttrium	3.75	12.47	Eutectic	[90]
Cerium	0.09	0.52	Eutectic	[90]
Neodymium	0.52	~3.00	Eutectic	[90]
Samarium	0.9	5.3	Eutectic	[90]
Gadolinium	4.53	23.49	Eutectic	[90]
Dysprosium	4.76	25.34	Eutectic	[90]
Erbium	6.91	33.8	Eutectic	[90]

SS = Solid solubility.

**Table 2:** The solubility product constant K_sp_ values at 25 °C [240,241].

Formula	K_sp_	Formula	K_sp_	Formula	K_sp_
Ag_3_PO_4_	8.89 × 10^−17^	Ag_2_CO_3_	8.46 × 10^−12^	Ag(OH)_2_	2.00 × 10^−8^
AlPO_4_	9.84 × 10^−21^	BaCO_3_	2.58 × 10^−9^	Al(OH)_3_	1.30 × 10^−33^
Ba_3_(PO4)_2_	3.40 × 10^−23^	CaCO_3_	3.36 × 10^−9^	Au(OH)_3_	5.50 × 10^−46^
Ca_3_(PO_4_)_2_	2.07 × 10^−33^	CdCO_3_	1.00 × 10^−12^	Bi(OH)_3_	6.00 × 10^−31^
Cd_3_(PO_4_)_2_	2.53 × 10^−33^	CuCO_3_	1.40 × 10^−10^	Ca(OH)_2_	5.02 × 10^−6^
Cu_3_(PO_4_)_2_	1.40 × 10^−37^	CoCO_3_	1.40 × 10^−13^	Cd(OH)_2_	7.20·10^−15^
Li_3_PO_4_	2.37 × 10^−11^	FeCO_3_	3.13 × 10^−11^	Ce(OH)_3_	1.60 × 10^−20^
Mg_3_(PO_4_)_2_	1.04 × 10^−24^	Li_2_CO_3_	8.15 × 10^−4^	Ce(OH)_4_	2.00 × 10^−28^
Ni_3_(PO4)_2_	4.74 × 10^−32^	MgCO_3_	6.82 × 10^−6^	Cu(OH)	1.00 × 10^−14^
AgCl	1.77 × 10^−10^	MnCO_3_	2.24 × 10^−11^	Ca(OH)_2_	2.2 × 10^−20^
AuCl	2.00 × 10^−13^	Nd_2_(CO_3_)_3_	1.08 × 10^−33^	Co(OH)_2_	5.92 × 10^−15^
AuCl_3_	3.20 × 10^−46^	NiCO_3_	1.42 × 10^−7^	Co(OH)_3_	1.60 × 10^−44^
CuCl	1.72 × 10^−7^	SrCO_3_	5.60 × 10^−10^	Cr(OH)_3_	6.30 × 10^−31^
		Y_2_(CO_3_)_3_	1.03 × 10^−31^	Cu(OH)_2_	2.20 × 10^−20^
		ZnCO_3_	1.46 × 10^−10^	Eu(OH)_3_	9.38 × 10^−27^
				Ga(OH)_3_	7.28 × 10^−36^
				Fe(OH)_2_	4.87 × 10^−17^
				Fe(OH)_3_	2.79 × 10^−39^
				Pb(OH)_2_	1.43 × 10^−20^
				La(OH)_3_	2.00 × 10^−19^
				Mg(OH)_2_	5.61 × 10^−12^
				Mn(OH)_2_	1.90 × 10^−13^
				Ni(OH)_2_	5.48 × 10^−16^
				Pr(OH)_3_	3.39 × 10^−24^
				Tl(OH)_3_	1.68 × 10^−44^
				Sn(OH)_2_	5.45 × 10^−27^
				Sn(OH)_4_	1.00 × 10^−56^
				Y(OH)_3_	1.00 × 10^−22^
				Zn(OH)_2_	3.00 × 10^−17^

## Glossary

CGRP	Calcitonin gene-related polypeptide-a
CREB1	CAMP-responsive element-binding protein 1
CT	Computed tomography
CF	Corrosion fatigue
HEPES	4-(2-hydroxyethyl)-1-piperazineethanesulfonic acid
H_2_	Hydrogen
HE	Hydrogen evolution
HERs	Hydrogen evolution reactions
HA	Hydroxyapatite
MEM	Minimum essential medium
NFATc1	Osteoclastogenesis-related gene
PBR	Pilling–Bedworth ratio
PLA	Polylactic acid
T6 treatment	Precipitation heat treatment
RE	Rare earth
REEs	Rare earth elements
ROS	Reactive oxygen species
SPD	Severe plastic deformation
SBF	Simulated body fluid
K_sp_	Solubility product constant
T4 treatment	Solution treatment
SCC	Stress corrosion cracking
TM	Transition metal
Tris	Tris (hydroxymethyl) aminomethane
